# Physico-Chemical Characteristics and Posterolateral Fusion Performance of Biphasic Calcium Phosphate with Submicron Needle-Shaped Surface Topography Combined with a Novel Polymer Binder

**DOI:** 10.3390/ma15041346

**Published:** 2022-02-11

**Authors:** Ruggero Belluomo, Inazio Arriola-Alvarez, Nathan W. Kucko, William R. Walsh, Joost D. de Bruijn, Rema A. Oliver, Dan Wills, James Crowley, Tian Wang, Florence Barrère-de Groot

**Affiliations:** 1 Kuros Biosciences BV, 3723 MB Bilthoven, The Netherlands; ruggero.belluomo@kurosbio.com (R.B.); nathan.kucko@kurosbio.com (N.W.K.); florence.de.groot@kurosbio.com (F.B.-d.G.); 2 Tissue Engineering Group, Biodonostia Health Research Institute, 20014 Donostia-San Sebastian, Spain; inazio.arriola@biodonostia.org; 3 Surgical and Orthopedic Research Laboratories, Prince of Wales Clinical School, UNSW Sydney, Sydney 2031, Australia; w.walsh@unsw.edu.au (W.R.W.); rema.oliver@unsw.edu.au (R.A.O.); d.wills@unsw.edu.au (D.W.); james.crowley@unsw.edu.au (J.C.); t.wang@unsw.edu.au (T.W.); 4 School of Engineering and Materials Science, Queen Mary University of London, London E1 4NS, UK

**Keywords:** synthetic bone graft, polymer, calcium phosphate, needle-shaped surface topography, putty, bioactivity, biodegradation, posterolateral spinal fusion

## Abstract

A biphasic calcium phosphate with submicron needle-shaped surface topography combined with a novel polyethylene glycol/polylactic acid triblock copolymer binder (BCP-EP) was investigated in this study. This study aims to evaluate the composition, degradation mechanism and bioactivity of BCP-EP in vitro, and its in vivo performance as an autograft bone graft (ABG) extender in a rabbit Posterolateral Fusion (PLF) model. The characterization of BCP-EP and its in vitro degradation products showed that the binder hydrolyses rapidly into lactic acid, lactide oligomers and unaltered PEG (polyethylene glycol) without altering the BCP granules and their characteristic submicron needle-shaped surface topography. The bioactivity of BCP-EP after immersion in SBF revealed a progressive surface mineralization. In vivo, BCP-EP was assessed in a rabbit PLF model by radiography, manual palpation, histology and histomorphometry up to 12 weeks post-implantation. Twenty skeletally mature New Zealand (NZ) White Rabbits underwent single-level intertransverse process PLF surgery at L4/5 using (1) autologous bone graft (ABG) alone or (2) by mixing in a 1:1 ratio with BCP-EP (BCP-EP/ABG). After 3 days of implantation, histology showed the BCP granules were in direct contact with tissues and cells. After 12 weeks, material resorption and mature bone formation were observed, which resulted in solid fusion between the two transverse processes, following all assessment methods. BCP-EP/ABG showed comparable fusion rates with ABG at 12 weeks, and no graft migration or adverse reaction were noted at the implantation site nor in distant organs.

## 1. Introduction

The biphasic calcium phosphate with submicron needle-shaped surface topography (BCP) investigated in this study has previously been demonstrated to be a promising solution for predictable spine fusion, circumventing the disadvantages of the gold standard autologous bone graft (ABG), i.e., limited availability, donor site morbidity, patient complications such as pain, secondary site infections, and subsequent risks of revision surgery [1,2,3,4]. In skeletally mature rabbit and sheep posterolateral fusion models, BCP showed fusion rates equivalent to ABG [5,6], and head-to-head comparison studies have shown an accelerated bone formation and superior fusion rate with needle-shaped surface topography compared to conventional grain-shaped surface topographies in dog muscle and sheep spine studies [7,8]. Recently, the first clinical case series reported successful fusion rates 12 months post-surgery near 97% (94 out of 97 levels) in the lumbar fusion cohort of 52 patients, and 93.8% (75 out of 80 levels) in the cervical fusion cohort of 25 patients [9].

The mechanism underlying these outcomes is associated with the needle-shaped submicron surface topography of BCP, which promotes the differentiation of macrophages, key players in the bone-biomaterial response, towards an anti-inflammatory M2 phenotype which has been associated with a favorable pro-healing mechanism [9,10,11,12,13]. The in vitro cell culture of human macrophages (THP-1 and primary cells) on the needle-shaped surface topography showed them skewing towards the favorable pro-healing M2 phenotype within the first 4 days as compared to a needle-free surface topography used as a control [9,13]. In high-throughput screening studies of surfaces consisting of needles, it was shown than only a subset of needles’ features promoted the favorable polarization towards M2 macrophages from murine and human origins [14,15]. The dimensional and density aspect of the needle features was also shown to influence adhesion, mechanics, and osteogenic differentiation from murine and human cells [16,17,18]. The submicron needle-shaped surface topography feature of BCP is, therefore, pivotal for predictable fusion and should be preserved when designing bone graft formulations suitable for the end-users.

The use of putty bone grafts is necessary to facilitate their handling and delivery during spinal fusion procedures and to control migration post-implantation. Putty bone grafts imply the introduction of a binder. From a design perspective, such a binder has an ancillary function by providing cohesion, moldability and resistance to irrigation when applied in bony sites during surgery. From a safety and performance perspective, the binder must be biocompatible, and it must not alter the intrinsic bone formation capacity of the bone graft upon manufacturing, sterilization, storage, and implantation [19,20,21,22]. Previously, we reported the biocompatibility and performance of a moldable BCP-Putty bone graft consisting of a water-soluble polymer that was found to be equivalent to BCP granules without a binder in a validated rabbit posterolateral fusion model [6]. To ease perioperative handling in the surgical theater, the BCP-EP bone graft was designed to obtain a ready-to-use formulation that is easier to dispense and to mold prior applications into the bone defect selected following a human-factor study.

BCP-EP comprises granules of a biphasic calcium phosphate with submicron needle-shaped surface topography embedded in a triblock copolymer binder consisting of a polyethylene glycol (PEG) central block and polylactide (PLA) blocks at each end (PLA-PEG-PLA). This so-called LEOL 400 binder provides cohesion and ease-of-use for application into the bone defect. Next to biocompatibility requirements, the LEOL 400 binder was specifically designed to preserve the submicron needle-shaped surface topography of BCP and its unique pro-healing mode-of-action for bone regeneration.

The focus of this study was, therefore, to evaluate the biological functionality of the BCP-EP in its finished configuration (i.e., sterilized), namely (1) the physico-chemical composition and in vitro degradation mechanism of BCP-EP, (2) the in vitro bioactivity of BCP-EP in simulated body fluid (SBF), and (3) the performance and biocompatibility of BCP-EP used as an autograft extender in 1:1 volume ratio with ABG (BCP-EP/ABG) in a rabbit Posterolateral Fusion (PLF) model compared to ABG alone.

## 2. Materials and Methods

### 2.1. Preparation of BCP-EP

BCP-EP (MagnetOs Easypack Putty; Kuros Biosciences B.V., The Netherlands) was manufactured by Kuros Biosciences B.V. (Bilthoven, The Netherlands). The BCP granules were prepared as described in Duan et al. from BCP powder mixed with a porogen, sintered, crushed, sieved to 1–2 mm granules, and finally subjected to hydrothermal treatment to obtain a needle-shaped submicron surface topography [7]. The LEOL 400 triblock copolymer was obtained by copolymerization of 80–90 wt% of PEGs ranging from 400 to 2000 Dalton (Da) with 10–20 wt% L-lactide monomer (LM) following ring-opening polymerization in the presence of Stannous Octoate under inert atmosphere at 130 °C. After 24 h, the LEOL 400 polymer was discharged and stored at −15 °C. The BCP-EP device was prepared by gentle penetration of molten LEOL 400 at 55 °C to fill solely the intergranular space of the BCP granules up to the top layer of the BCP granules in an open-ended syringe. The resulting BCP-EP device consisting of >95 vol% of BCP granules was sterilized by gamma irradiation (25–40 kGy) and used for all subsequent in vitro and in vivo testing.

### 2.2. Characterization of BCP-EP

BCP granules extracted from the BCP-EP were subjected to X-ray Diffractometry (XRD) and Scanning Electron Microscopy (SEM) analysis (all the analyses were performed in triplicates, *n* = 3). XRD was performed to determine the phase composition using internal calibration, at a scan speed of 1deg/min, sampling of 0.01deg, and a scan range of 25–45 degrees (Rigaku Miniflex II). SEM was performed for surface topography analysis at several magnifications ranging from 500× to 8000× (JEOL JSM-5600) after gold-sputtering (JEOL JFC-1300). Three images per sample were used to determine needle size (roughly 100 needle-like structures per image) at 8000× magnification using the AxioVision software (Carl Zeiss Microimaging, Inc., Jena, Germany).

LEOL 400 extracted from the BCP-EP was analyzed by 1H-Nuclear Magnetic Resonance (1H-NMR) and Fourier-Transform Infrared Spectroscopy (FT-IR). 1H-NMR was performed to determine the structure and composition of the triblock copolymer in deuterated chloroform (CDCl3) (Bruker, 400 MHz, NMR A). The 1H-NMR data were processed in-house using ACD/NMR Processor software (Academic version 12.0, ACD Labs, Inc., Toronto, ON, Canada) to determine polymer composition and molecular weight. The molar fractions for PLA, PEG, and LM % in the triblock copolymer were calculated from the contributing protons of each chemical species and their respective intensities, i.e., 4 contributing protons for each ethylene oxide unit contained in PEG and for each lactide unit contained in PLA, and 8 protons for LM. LEOL 400 molecular weight, were calculated using the same intensities of each chemical species, the number of repeating units in the polymer, and their respective molar mass, i.e., ethylene oxide (44 g/mol) and lactide monomer (144 g/mol). The Jasco FT-IR-4100 equipment (JASCO Co., Tokyo, Japan) FT-IR was used to determine the identity of the triblock copolymer using a scan range of 4000–500 cm^−1^, an accumulation of 50, a resolution of 4.0 cm^−1^, and a TGS detector (FT-IR-4100, JASCO).

### 2.3. In Vitro Degradation of BCP-EP

The in vitro degradation mechanism of LEOL 400 from BCP-EP was assessed according to ISO 13781 guidelines [23]. A quantity of 1 g of BCP-EP was incubated in 60 mL of 0.01 M Phosphate Buffered Saline at pH = 7.3 (PBS, Gibco Thermo Fisher) in a shaking water bath at 37 °C. The study was carried out for a total of 14 days. pH measurements were taken at timepoints 0, 1, and 6 h as well as 1, 7, and 14 days and compared to negative controls (2 PBS reference samples). One sample was collected after 1 h, and 1- and 14-days incubation, and immediately freeze-dried to stop the reaction. The ceramic and polymeric phases were separated using chloroform prior to characterization as described above (*n* = 1 per time point).

### 2.4. Bioactivity Testing of BCP-EP in SBF

The in vitro mineralization potential of BCP-EP was assessed in Simulated Body Fluid (SBF) [24,25]. A clean glass beaker was filled with 750–800 mL of RX-water and stirred at 36.5 °C. Reagents were added in strict order according to Campion et al., 2013 [25]. The solution was then sterilized using a Nalgene Rapid-Flow Vacuum Filter Unit, cooled down, and stored at 4 °C until use.

Two blocks of approximately 0.5g BCP-EP were collected under aseptic conditions and immersed individually in 200 mL SBF at 37 °C in an agitated water bath. SBF was refreshed every 2–3 days until day 10. At days 2, 4, 7, and 10, granules released in the solution were collected, rinsed twice in RX-water, and placed in a drying oven at 80 °C overnight. At least 5 granules were imaged per time point at 500×, 2000×, and 8000× magnification to assess mineralization by SEM. A negative control (crushed particles of Polystyrene, CellStar) and a positive control (Bioactive Glass component from Vitoss BA) underwent the same treatment and SEM analysis.

### 2.5. Twelve-Week Implantation Study of BCP-EP/ABG in a Rabbit Posterolateral Fusion Model

The in vivo performance of BCP-EP was evaluated as an autograft extender (BCP-EP/ABG in a 1:1 volume ratio) in a validated skeletally mature rabbit Posterolateral Fusion (PLF) model and compared to a positive control autologous bone graft (ABG) [6,26,27,28].

#### 2.5.1. Preparation of Graft Materials

The iliac crests were exposed from the caudal portion of the midline incision [28]. Cortico-cancellous bone was collected from both iliac crests using a Miltex Rongeur. The volume and weight of the ABG particles (<5 mm in size) were measured on a laboratory balance and a 3 mL syringe (tip removed).

The BCP-EP/ABG group was prepared by combining 1 cc (~0.8 g) of ABG particles with 1 cc of BCP-EP. Volumes of 2 cc of ABG and BCP-EP/ABG were prepared for implantation on each side of the spine (4 cc total per level).

#### 2.5.2. Animal Model and Surgical Procedure

Upon approval by the Institutional Animal Care and Ethics Committee (ACEC approval 20/62A, UNSW, Australia), twenty skeletally mature female New Zealand White Rabbit were subjected to single-level bilateral posterolateral intertransverse spinal fusion surgery at L4–5. Radiographic analysis of the closure of the growth plates located in the proximal tibia was used to assess skeletal maturity [28]. A combination of midazolam (5 mg/mL at 0.3–0.5 mg/kg) and buprenorphine (0.326 mg/mL at 0.03–0.05 mg/kg) was injected intramuscularly to sedate the animals prior to the operation. Anesthesia was performed and maintained through continuous inhalation of isoflurane (2–3%) and oxygen. Once anesthetized, blood was harvested from either the jugular or saphenous vein prior to surgery to assess animal health, then the surgeon incised the skin of the rabbit at L3–L5 levels. The intertransverse membrane and the transverse processes were exposed by separating the intermuscular plane between the multifidus and longissimus muscles in a blunt manner. Decortication was performed on the host bone between the levels using a pneumatic burr (Midax Rex) and an M8 matchstick burr. The transverse processes were meticulously decorticated at 10 mm from the vertebral body and pars to a point where the surgeon could visualize bleeding bone beds [28].

The animals were randomly divided into two groups: (1) ABG and (2) BCP-EP/ABG. In the paraspinal bed between the L4–5 transverse processes, each group received a total volume of 2 cc graft material. The muscle layers were allowed to return to their natural position after implant insertion, and the fascial incisions were closed with 3-0 absorbable sutures. For 2–3 days after the surgical operation, the pain was treated with anti-inflammatory and non-steroid medicine (Carprofen, 50 mg/mL, dose 2–4 mg/kg). The animals were kept in pens, fed ad libitum, and checked daily for the first seven days after the operation, then weekly after that. Animals were euthanized after 3 days (*n* = 2/group) and 12 weeks (*n* = 8/group) for analysis.

#### 2.5.3. Radiographical Fusion by X-rays and Micro-CT

For all animals, post-sacrifice, faxitron and digital plates (AGFA CR MD4.0 Cassette) were used to radiograph the extracted spines (32 kV, 25 s), and micro-CT (Inveon, Siemens Medical, Malvern, PA, USA) was used to obtain high-resolution (40 µm) images of the spinal fusions in three planes (axial, coronal, and sagittal). The digital photos were processed using an AGFA Digital Developer and workstation (AGFA CR 75.0 Digitiser Musica, AGFA, Germany). Medical viewer ezDICOM was used to convert DICOM data. Analysis of the Faxitron radiographs and micro-CT was conducted in a blinded manner by two independent observers in the posteroanterior and in the coronal, axial, and sagittal planes to assess the quality of the fusion between the two transverse processes using the Lenke four-point grading scale [29]. A grade of A was assigned to a fusion that was determined to be solid, with a bilateral strong bridging bone. A fusion that was assessed to be likely solid with a unilateral strong bridging bone and a contralateral thin fusion mass received a grade B. A fusion that was assessed to be likely not solid with a thin unilateral fusion mass and possible pseudarthrosis on the contralateral side received a grade C. A fusion that was assessed to be obviously not solid with thin fusion masses bilaterally and apparent pseudarthrosis or bone graft disintegration received a grade D. Based on film evaluation, the proportion of successful fusions for each grade was determined.

#### 2.5.4. Fusion by Manual Palpation Testing

Fusion by manual palpation testing was performed on the spines immediately after harvest to determine lumbar spine stability. All animals for all time points were subjected to manual palpation following Boden et al. [26]. In a blinded manner, lateral bending and flexion/extension analysis was examined at the treated segment and compared to the distal and proximal motion segments by two blinded observers. At L4–5, the motion segments were rated as fused (rigid, no observable movement at the disc space) or not fused (not rigid, movement detected at the disc space).

#### 2.5.5. Histological Fusion and Histomorphometry

After the above evaluations, all spines were fixed for at least 96 h in a 0.145 M PBS with 10% formalin. Using a hacksaw, the spines were sliced in the sagittal plane through the center of the vertebral body. One side underwent processing for decalcified paraffin histology to assess overall tissue response and fusion. The other side underwent processing for undecalcified polymethyl methacrylate (PMMA) histology for quantitative histomorphometry.

- Decalcified histology: Decalcification of the spines was conducted in 10% formic acid at room temperature for 3 to 4 days. The spine was sectioned in four blocks of ~3 mm prior to immersion in paraffin. Three sections of 5 microns were obtained from each paraffin block using a Leica microtome (Leica Microsystems Pty Ltd., North Ryde, NSW, Australia) and were subsequently stained with hematoxylin and eosin (H&E). An Olympus light microscope (Olympus, Japan) was used to analyze the stained sections in a blinded manner. The qualitative assessment of tissue response, signs of graft resorption, new bone formation, the presence of inflammatory cells or tissue necrosis, development of the marrow space, and bony fusion across the two transverse processes were all included in the examination. On the spine of a Rallis’ Tetrachrome [30], staining was used to observe bone maturation on decalcified paraffin histology.
- Undecalcified histology: The spines were dried with ethanol series prior to embedding in PMMA. Then, 15-micron-thick sections of the sagittal plane obtained through a Leica SP1600 saw microtome (Leica Microsystems Pty Ltd., North Ryde, NSW, Australia) were stained with methylene blue (1% in 0.1 M borax buffer, pH 8.5) and basic fuchsin (0.3% in water). All PMMA blocks were cut into three sections, with a space of 2 mm between each, to analyze the complete fusion mass. Images at low magnification were finally used for histomorphometrical analysis.
- Histomorphometry: For a quantitative analysis, three images at low magnification (1.25×, 1 mm scale bar) from the two transverse processes and the fusion center were used. An investigator blinded to the study groups used a polygon approach to establish the region-of-interest (ROI), and graft material or bone mineralized tissue was assessed by pixel color and morphological evaluation using established techniques [6]. Each fusion area was calculated as a ROI percentage, and a mean value was calculated based on the three PMMA sections.
- Distant organ analysis: The heart, liver, lungs, kidney, spleen, lymph nodes, pancreas, adrenal gland, thyroid gland, pituitary gland, and ovaries were harvested. A portion of each tissue was similarly processed for paraffin histology, H&E staining, and routine histopathological review for signs of any adverse reactions.

#### 2.5.6. Statistical Analysis

Statistical analysis was conducted using SPSS for Windows (SPSS, Chicago, IL, USA). A Kruskal–Wallis analysis of variance was used to examine the data of the fusion grading. When applicable, data from biomechanical testing and histomorphometry were evaluated using a 1-way analysis of variance followed by a Games–Howell post-hoc test. The threshold for statistical significance was fixed at *p* < 0.05.

## 3. Results

### 3.1. Characterization BCP-EP

The XRD spectrum of the BCP granules extracted from BCP-EP showed the presence of beta-tricalcium phosphate (TCP) and hydroxyapatite (HA) phases (composition of 65–75% β-TCP and 25–35% HA). No other phases were detected. SEM images of BCP granules from BCP-EP showed a homogeneous submicron needle-like surface structure with an average needle size of approximately 0.60 µm.

**Figure 1:**
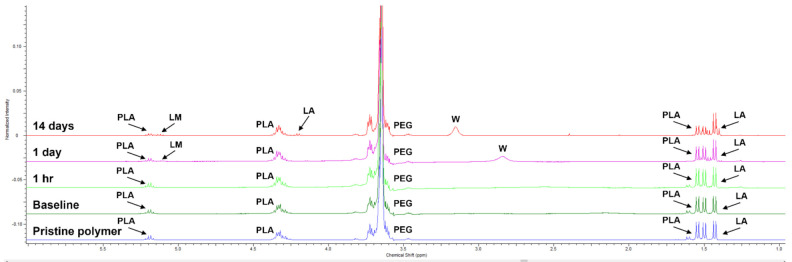
1H-NMR spectra of pristine LEOL 400 after synthesis (pristine polymer), extracted from gamma-sterilized BCP-EP (baseline), and collected after immersion of BCP-EP in 0.01 M PBS at 37 °C for 1 h, 1 day and 14 days. The characteristic chemical shifts are highlighted with reference to their compounds. LA: lactide; PLA: polylactide; LM: lactide monomer; W: water.

**Figure 2:**
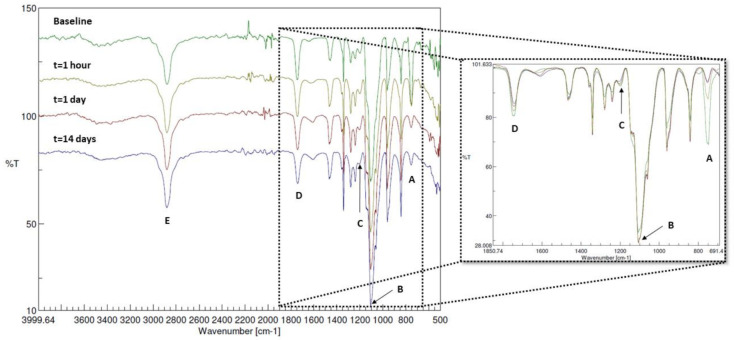
FT-IR spectra of the collected polymer prior to (baseline, t = 0, green) and after immersion in PBS for 1 h (yellow), 1 day (red) and 14 days (purple), with a zoomed in overlayed spectrum between 1850 and 700cm^−1^. The letters correspond to the different characterizations of the PEG or PLA, whereby (A): C=O (bend) backbone of PLA; (B): C-O-C (stretch) of PEG; (C): ester bonds (stretch) between LA units in PLA blocks and PLA-PEG; (D): C=O (stretch) of PLA; (E): C-H (stretch) from both PLA and PEG.

1H-NMR and FT-IR analysis of the LEOL 400 extracted from BCP-EP (baseline) shown in Figure 1 and Figure 2 confirmed the identity and composition of the polymer (PLA-PEG-PLA). In Figure 1, the 1H-NMR spectrum showed the characteristic chemical shift at 3.40–3.90 ppm related to the backbone of the PEG polymer (methylene group, -CH_2_), and at 5.10–5.30 and 1.40–1.65 ppm related to the PLA backbone (methyl and methine groups, -CH and -CH_3_, respectively). In the 1.40–1.65 ppm PLA region, two sets of resonances at differing chemical shifts at 1.40–1.50 ppm and 1.50–1.65 ppm were observed, suggesting that the PLA methyl protons exist in different chemical environments in proximity to the PEG groups or that there are differences in the packing of adjacent PLA chains [31]. The absence of shifts at 1.65–1.90 and 4.80–5.15 ppm (associated with potentially unreacted lactide monomer) confirms the completion of the copolymerization process [32,33]. The molar fraction was determined to be 87% PEG and 13% PLA based on the integrals of the respective 3.40–3.90 ppm and 1.40–1.65 ppm shift regions. A molecular weight of 1708 Da was determined for LEOL 400 extracted from BCP-EP (baseline, Table 1). There were no significant differences with the pristine polymer directly after synthesis. Identical shifts and peak shape characteristics, molar fractions, and PLA:LA molar ratios for sets of lactide resonances at 1.40–1.50 ppm and 1.50–1.65 ppm and a highly similar molecular weight of 1702 Da were determined, suggesting the stability of LEOL 400 polymer upon gamma-sterilization in the 25–40 kGy range (Figure 1, Table 1).

The FT-IR spectrum of the LEOL 400 extracted from BCP-EP (baseline) showed peaks characteristic of PEG, PLA, and PEG-PLA bonds. The PEG block was identified by the bands at 2885, 1450-1290, 960, and 840 cm^−1^ corresponding to the CH_2_ bond of the PEG backbone and by the bands located in the 1160–1000 cm^−1^ region corresponding to the C-O-C bond of the PEG backbone [32,33,34]. The bands related to the PLA block were identified by the bands located at 750 and 1760 cm^−1^, corresponding to the C=O bond, and by the band located at 1190 cm^−1^, corresponding to C-O-C bonds in the PLA block and to a bond between PLA and PEG blocks [32,33,35]. The band located at 2885cm^−1^ is typical for the C-H stretching vibration of -CH_3_ and -CH- groups corresponding to both PLA and PEG [32,33,35].

### 3.2. In Vitro Degradation of BCP-EP

The initial pH of 7.3 at the start of the experiment decreased gradually to 6.5 after 1 h, 6.0 after 1 day, 5.5 after 6 days, and 5.0 after 10 days of immersion, indicating the production of lactic acid resulting from PLA hydrolysis and subsequent reduction in pH. 1H-NMR spectra of the polymer extracted from BCP-EP confirmed the hydrolysis of PLA. Figure 1 shows the 1H-NMR spectra of the LEOL 400 polymer at t = 0 (baseline) and after 1 h, 1 day, and 14 days of incubation in PBS. From 1 h onwards, changes were observed in the shift regions corresponding to the PLA block. Overtime, the shifts at 1.50–1.65 ppm and 4.20–4.45 ppm corresponding to polylactide molecules decreased, while there was an increase in the shifts at 1.40–1.50 ppm corresponding to the higher presence of lactic acid molecules. The drop in the shift at 5.15–5.30 indicating the presence lactic acid and lactide oligomers, and the appearance of the shift at 4.20–4.45 ppm corresponding to lactic acid oligomers further demonstrated the hydrolysis of the PLA molecules. The quantification overtime of the LA/PLA ratio increased from 0.33 at baseline to 0.80 after 14 days. Concomitantly, the average molecular weight of the residual polymer decreased from 1708 Da to 1591 Da after 14 days (Table 1). No changes in the 3.40–3.90 ppm region corresponding to the PEG molecule, nor new PEG derived molecules, could be detected by NMR, thereby suggesting that the PEG molecules remained unaltered during the LEOL 400 degradation mechanism in vitro.

**Table 1:** Molecular weight in Dalton (Da) and LA/PLA ratio of LEOL 400 after synthesis (pristine), extracted from gamma-sterilized BCP-EP (baseline), and collected after immersion of BCP-EP in 0.01M PBS at 37 °C for 1 h, 1 day, and 14 days.

Immersion Time	Pristine	Baseline	1 h	1 Day	14 Days
Average Mn (Da)	1702	1708	1715	1685	1591
LA/PLA ratio	0.33	0.33	0.33	0.50	0.80

Figure 2 shows the FT-IR spectra of the LEOL 400 polymer at t = 0 (baseline) and after 1 h, 1 day, and 14 days of incubation in PBS. From 1 h onwards, the A, C, and D bands corresponding to PLA at 750, 1190, and 1760 cm^−1^ decreased in intensity overtime when compared to the dominant band at 1100 cm^−1^ corresponding to the C-O-C bond for PEG (band B in Figure 2) as a result of PLA hydrolysis at the C-O-C ester bond. The PEG bands located at 2885, 1450–1290, 960, 1320–1000, and 840 cm^−1^ did not change in configuration or intensity, indicating that the PEG block remained unaltered during the PLA hydrolysis. FT-IR confirmed that LEOL 400 degraded in vivo by means of hydrolysis of the PEG-PLA bonds and PLA molecules into lactic acid oligomers, while PEG molecules remained unaltered.

The XRD spectra and SEM images of BCP granules extracted after all incubation time points showed a comparable pattern consisting of TCP and HA (composition of 25–35% HA and 65–75% β-TCP, and no presence of new CaP phases), and no changes in needle surface structure. These results indicated that LEOL 400 hydrolyses readily in physiological conditions, resulting in unaltered PEG molecules, lactide species, and lactic acid that do not affect the physio-chemical characteristics of BCP granules.

### 3.3. Bioactivity

**Figure 3:**
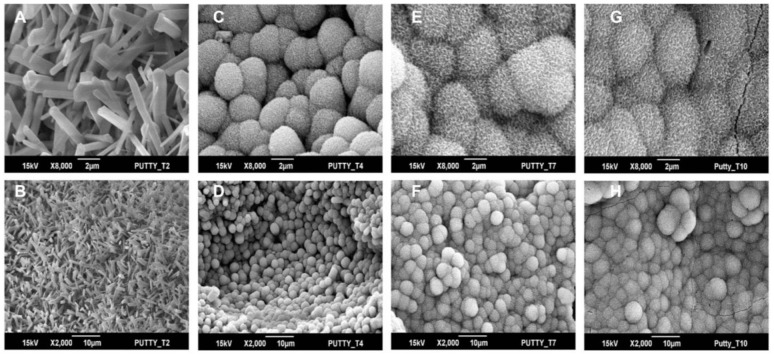
SEM images of surface mineralization on BCP-EP after (**A**,**B**) 2 days, (**C**,**D**) 4 days, (**E**,**F**) 7 days, and (**G**,**H**) 10 days immersion in SBF. Images were taken at 8000× (top panel) and 2000× (bottom panel) magnification. The needle-shaped surface topography at day 2 was identical to the baseline prior to immersion in SBF and identical to BCP granules prior to mixing with LEOL 400.

Surface mineralization observed on BCP-EP over time is shown in Figure 3. From day 4 onwards, globules of bone-like mineral hydroxy-carbonated apatite (HCA), of 1–5 µm in diameter, could be seen growing on the needles of BCP granules [25,26]. From day 7, the surface of the BCP granules was entirely covered by HCA globules of 5–10 µm in diameter. At 8000× magnification, the morphology of the HCA consisting of a rod-like structure of ~0.3 µm in length and 0.05 µm in diameter was comparable between BCP-EP and the positive control Bioactive Glass. These results confirm that the LEOL 400 hydrolysis does not impact the bioactive potential of BCP granules.

### 3.4. Twelve-Week Implantation Study of BCP-EP/ABG in a Rabbit Posterolateral Fusion Model

Surgery was completed in all animals with no complications. All animals ambulated normally during the entire study duration. The fusion assessments conducted at 12 weeks by means of radiography, micro-CT, manual palpation, and histology for BCP-EP/ABG and ABG are reported in Table 2.

#### 3.4.1. Radiographical Fusion by X-ray and Micro-CT

**Figure 4:**
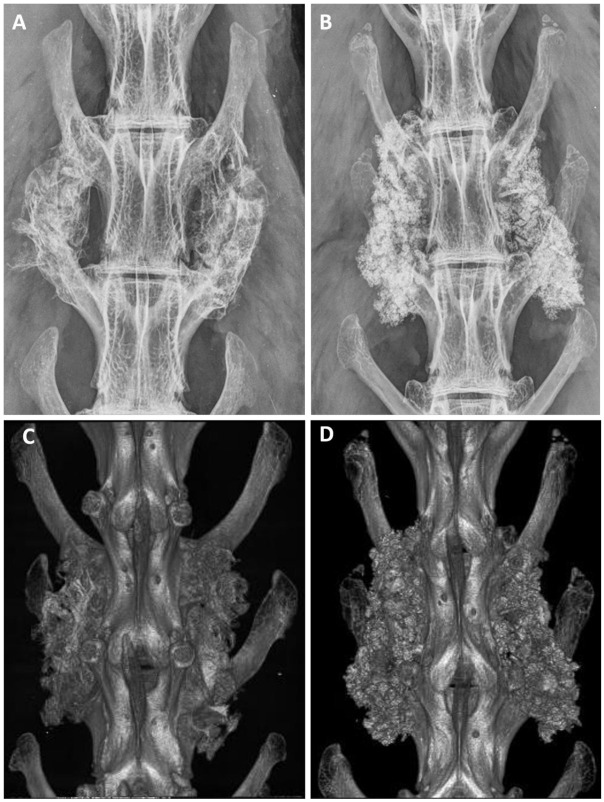
Representative examples of Faxitron radiographs taken after 12 weeks for (**A**) ABG and (**B**) BCP-EP/ABG. Representative examples of 3D micro-CT reconstructions taken after 12 weeks for (**C**) ABG and (**D**) BCP-EP/ABG. The Faxitron radiographs and micro-CT reconstructions show the presence of graft material bridging the intertransverse process space of the treated levels in all groups.

After euthanasia, anteroposterior radiographs were analyzed to evaluate the implantation. The analysis by radiography and micro-CT revealed successful implantation in all animals, and no adverse bony reactions or graft migration (Figure 4). The grading distribution according to the Lenke scale was comparable between ABG and BCP-EP/ABG with no statistical difference. A high occurrence of Grade A was observed at 12 weeks for both groups through radiographic analysis and micro-CT scans, indicating the presence of a solid bilateral fusion at this timepoint. The fusion rates were 62.5% and 75% for BCP-EP/ABG and ABG, respectively, according to both radiographic and micro-CT analysis.

#### 3.4.2. Fusion by Manual Palpation Testing

Fusion evaluation through manual palpation showed consistent and comparable bilateral fusion in the ABG and BCP-EP/ABG groups with no statistical differences. Both groups reported a bilateral fusion rate of 75% at 12 weeks after implantation.

#### 3.4.3. Histological Fusion and Histomorphometry

**Figure 5:**
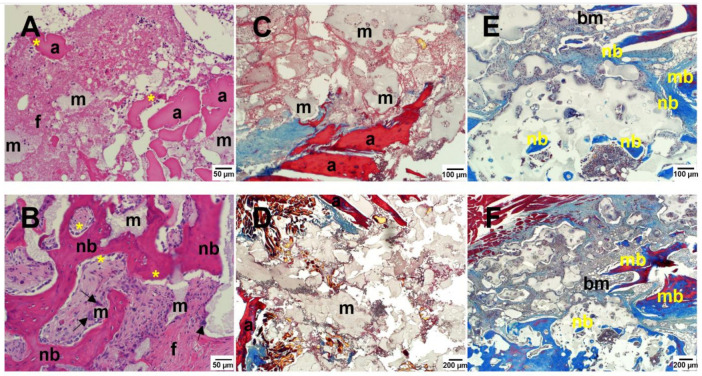
H&E staining of the transverse processes at (**A**) 3 days and (**B**) 12 weeks shows that granules released by BCP-EP are in direct contact with cells, including multinucleated cells (black arrow) and extracellular matrix. Osteoblasts (yellow asterisks) were observed to deposit new bone matrix. Tetrachrome staining at (**C**,**D**) 3 days and (**E**,**F**) 12 weeks showed the progressive formation and maturation of novel bone and bone marrow spaces. No adverse reaction was noted. Legend: m: calcium phosphate material; a: autograft particles; b: bone; nb: new bone; mb: mature bone; f: fibrous tissue; bm: bone marrow.

Histological evaluation was performed at low and high magnification. ABG histology at day 3 was characterized by autologous bone chips evenly distributed in the implantation sites, with inflammatory cells occasionally observed and blood clots seen. Similarly, the BCP-EP/ABG histology was characterized by autologous bone chips and BCP granules evenly distributed in the implantation sites, with inflammatory cells occasionally seen in comparable amount and morphology. Cells and extracellular matrix were observed in direct contact with the BCP granules at its surface and in the pores, showing that the LEOL 400 polymer had resorbed in vivo, allowing for the exposure of the BCP granules in less than 3 days (Figure 5A,C,D). ABG histology at week 12 was characterized by less residual ABG and continued new woven bone formation and remodeling with developing marrow spaces, resulting in new bone and fibrous tissue formation. Histology of BCP-EP/ABG at 12 weeks was characterized by normal bone healing and remodeling, resulting in new bone formation and resorption of BCP granules. New bone, maturing bone marrow, osteoblasts, multinucleated cells, and tissues were in direct contact with the BCP granules (Figure 5B,E,F). A similar tissue response was found for both groups, indicating no adverse reactivity upon implantation of BCP-EP/ABG, confirming the local biocompatibility of the BCP component and showing the local biocompatibility of the BCP-EP and its LEOL 400 component.

High-magnification micrographs revealed that the BCP granules were in direct contact with cells and tissues, confirming that LEOL 400 polymer and its degradation product did not impact the local tissue response.

**Figure 6:**
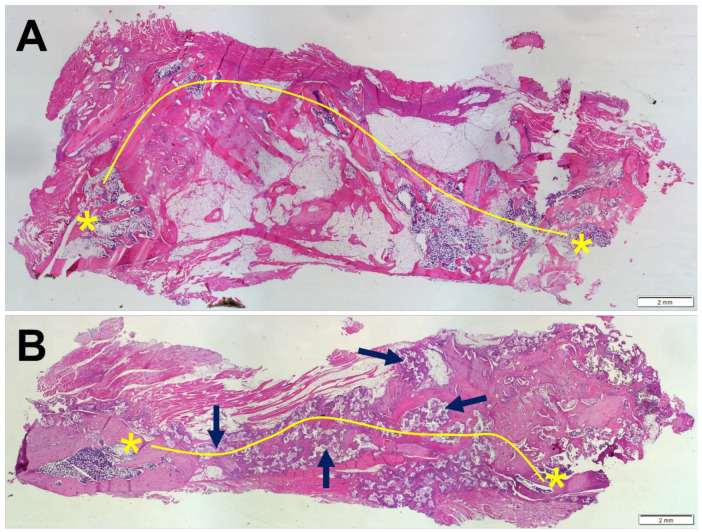
Overview of the histological panels (H&E) of the fusion masses present in the spine of (**A**) ABG and (**B**) BCP-EP/ABG at 12 weeks after surgery. Both fusion masses were evaluated as fused since a continuous bone mass (yellow line) was present between the transverse processes (yellow asterisk). The presence of BCP granules in the newly formed bone was confirmed in the BCP-EP/ABG group (black arrows).

Fusion was confirmed when a bony bridge connecting the transverse processes was formed (Figure 6). Paraffin H&E histology grading revealed that 62.5% and 75% of animals showed bilateral fusion in ABG and BCP-EP/ABG, respectively, at 12 weeks post-operation.

**Figure 7:**
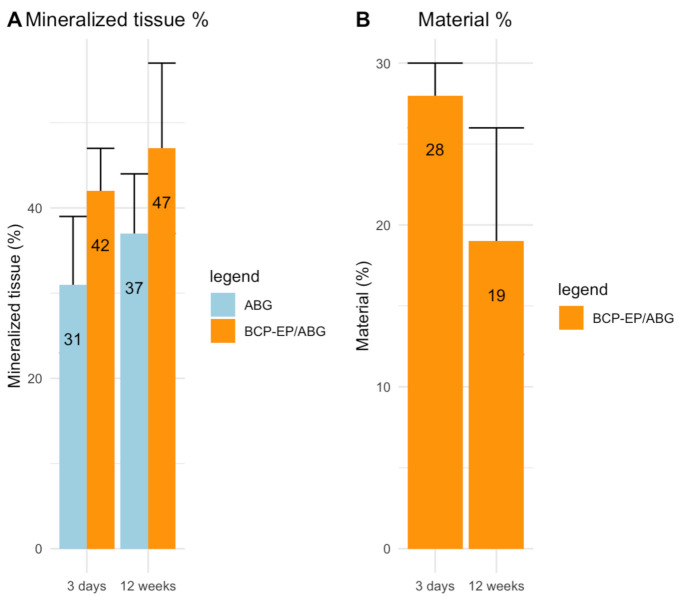
Graphs of the histomorphometrical analysis after 3 days and 12 weeks of implantation of ABG (light blue) or BCP-EP/ABG (orange): (**A**) mineral tissue percentage and (**B**) material resorption. Mineralized tissue increased overtime and resorption of BCP-EP was observed. Data are shown as mean ± sd. No statistical difference was observed between ABG and BCP-EP/ABG at week 12.

The histomorphometrical analysis was consistent with previous findings showing that new bone formation was accompanied by calcium phosphate granule resorption (Figure 7). The bone mineral percentage reached at 12 weeks was 37% and 47% for ABG and BCP-EP/ABG, respectively. No statistical difference between ABG and BCP-EP/ABG was observed at 12 weeks in terms of bone formation. This result shows an equivalent bone-forming property between ABG and BCP-EP/ABG. Resorption of BCP granules had partially taken place after 12 weeks of implantation.

The histopathological observations of the H&E-stained organ sections for both groups confirmed the absence of any systemic effect related to the implant material, confirming the biocompatibility of the BCP component and showing the biocompatibility of the BCP-EP and its LEOL 400 component.

## 4. Discussion

This study evaluated BCP-EP bone graft in vitro and in vivo, in particular the effect of the triblock PLA-PEG-PLA polymer (LEOL 400) designed to be used as a binder to provide improved handling characteristics to the BCP granules without impacting the bone healing performance. In vitro, the hydrolysis of LEOL 400 and its degradation products was analyzed by pH, FT-IR, and 1H-NMR. The submicron needle-shaped surface topography of BCP granules and the ability to support bone-like apatite formation upon LEOL 400 hydrolysis was assessed after immersion in SBF solution. In vivo, BCP-EP was compared as an autograft extender against autologous bone graft in a validated PLF model in rabbits after 3 days and 12 weeks of implantation. Outcomes were determined by an extensive range of assessment methods, including manual palpation, radiography, micro-CT, histology, and histomorphometry, to assess fusion performance and biocompatibility.

The characterization of the LEOL 400 component in BCP-EP showed a triblock PLA-PEG-PLA copolymer as demonstrated by FT-IR where typical PEG, PLA, and PEG-PLA chemical bonds could be clearly identified. The resulting copolymer has a molecular weight of 1708 Da consisting of 87% PEG and 13% PLA as determined by 1H-NMR. The hydrophilic PEG central block is bound at both ends to short PLA blocks. The dominant contribution of PEG together with a relatively low molecular weight of LEOL 400 are responsible for the ease of moldability and the desired solubility of BCP-EP bone graft. Compared to the pristine LEOL 400 polymer, 1H-NMR analysis showed no differences in identity, molar fractions, or molecular weights. Chain scission could not be observed as published by other groups that studied the effect of gamma-irradiation on PEG or PLA homopolymers and PLA/PEG copolymers [36,37,38].

The stability of LEOL 400 polymer upon gamma-irradiation is most probably related to the significantly smaller molecular weight of the copolymer and the higher PEG contribution compared to copolymers studied by Garric et al. and Dorati et al. (>150-fold) [37,38]. PEGs are subjected to crosslinking and chain scission upon gamma irradiation; the mechanisms are highly dependent on the atmospheric exposures and the changes are more pronounced with higher irradiation doses [36]. Yet, such mechanisms observed by Pucic et al. on PEGs ranging between 400 kDa and 8000 kDa exposed to a minimum of 50 kGy are unlikely to occur for LEOL 400 due to the significantly lower PEG molecular weight and irradiation range. Furthermore, the highly limited surface exposure of BCP-EP due to the LEOL 400 waxy texture combined with BCP granules in a closed syringe may further prevent oxidization [36]. While other techniques could be used to study the effect of gamma-sterilization on LEOL 400 polymer with greater precision, our results showed a polymer that remained stable upon gamma-irradiation.

The physico-chemical changes of BCP-EP occurring in vitro over 14 days at 37 °C consisted of the rapid hydrolysis of LEOL 400 polymer into unaltered PEG molecules, lactide species, and lactic acid. This hydrolysis took place at the PEG-PLA ester bond in less than one hour because of the high content of hydrophilic PEG, and the short PLA segments in the LEOL 400 formulation. 1H-NMR and FT-IR analyses demonstrated that the PEG blocks remained unaltered while the PLA blocks hydrolyzed further, in accordance with other in vitro degradation studies on PLA-PEG-PLA triblock copolymers [39]. The gradual hydrolysis of PLA into lactic acid and lactide oligomers was confirmed by all analytical methods. After 14 days of immersion in PBS, the PLA shifts had significantly decreased due to their breakdown into lactic acid and lactide oligomers. Nevertheless, hydrolysis was not complete due to the acidic conditions of our in vitro experiments. Research on PLA hydrolysis showed that PLA’s degradation is pH-dependent, being maximal under acidic (pH = 1–3) and neutral (pH ≈ 7–7.5) environments, and minimal at pH = 4.5 [40,41]. This mild acidic pH observed in vitro is unlikely to occur in vivo. Fluid circulation and its three main buffering systems (carbonic acid bicarbonate, phosphate, and proteins) can actively prevent local acidification [42]. Further, bone mineral acts as a local buffer by releasing Ca^2+^ into the media when the H^+^ concentration surpasses the buffering capacity of the aforementioned mechanisms [42]. Therefore, in vivo, the local pH upon implantation of BCP-EP is expected to stay neutral, at which the PLA hydrolysis into lactide and lactic acid will be complete.

The rapid clearance of LEOL 400 polymer, the absence of any local adverse reaction, and the absence of residual PLA or PEG was further confirmed by histology after 3 days and 12 weeks of implantation. At the earliest implantation time point of 3 days, tissue penetration throughout the graft and the direct contact between tissues was evident, with cells in contact with granules through the pores further confirming that the in vivo resorption of the LEOL 400 polymer occurs readily after implantation and is complete 3 days post-implantation without adverse local reactions, as demonstrated by histology. Throughout the implant, loose connective tissue and cells were observed to be in direct contact with the BCP granules. This demonstrated that LEOL 400 had been cleared by this time, allowing cell and tissue infiltration throughout the implanted graft material. Excessive inflammatory reaction was not observed, indicating that LEOL 400 and its degradation products did not elicit an adverse tissue response in vivo. The absence of any adverse tissue response and any excessive inflammatory response was further confirmed 12 weeks post-surgery. The BCP granules were found to be in direct contact with bone tissue, bone marrow, and other tissues, with no fibrous encapsulation. Cell-mediated resorption of the ceramic material was observed, similar to the phagocytosis mechanism observed on the BCP granules implanted without binder in the same model [6]. There was no significantly different local reactivity compared to the ABG group. This group, consisting of autologous bone collected from the iliac region of the host rabbit, presented the advantage of providing a baseline to assess any materials’ effects.

The gross observation of the distant organs at necroscopy combined with histopathological observations after 3 days and 12 weeks of implantation of BCP-EP/ABG confirmed that the LEOL 400 degradation products did not accumulate nor elicit toxicity. The metabolism, excretion, and non-toxicity of lactide oligomers, monomers, and lactic acid resulting from the hydrolysis of PLA blocks have been widely proven to be non-toxic for several decades. They can be rapidly excreted via the liver, lungs, and kidneys [43,44]. Similarly, the metabolism, excretion, and non-toxicity of PEG molecules of a wide molecular weight range has been widely established for all administration routes. The urinary clearance route dominates for all PEGs up to 190,000 Da molecular weight [45,46]. Similar to the observations made locally at the bone implantation site, there was no significantly different systemic reactivity compared to the positive control ABG group, thereby excluding any adverse systemic reaction related to BCP-EP and its degradation products.

The lack of adverse reaction based on local and systemic histological observations further confirms the safe use of the stannous octoate catalyst, one of the most widely known and used catalysts for PLA polymerization, which is approved by the FDA for human use and commonly used for other approved bone contacting devices [47].

In terms of performance, LEOL 400 polymer and its hydrolysis in vitro did not affect the submicron needle-shaped surface structure or phase composition of the BCP granules, as demonstrated by SEM and XRD. Additionally, LEOL 400 did not impact the bioactivity potential of the granules as demonstrated by the gradual and homogeneous bone-like mineralization of the BCP granules upon immersion in SBF. In vivo, the BCP-EP/ABG supported bone formation over time, leading to solid fusion masses between the transverse processes after 12 weeks. The results of fusion assessment conducted by means of manual palpation, radiography (X-ray, micro-CT), and histology revealed equivalent fusion rates compared to the positive control ABG group at all time points, with satisfactory fusion rates after 12 weeks corresponding to the literature [6,26], and comparable to BCP granules implanted in the absence of a polymeric binder [6]. The variation in fusion rate inherent to each method observed in this study is in line with previously published results in this rabbit PLF model [6,26,48]. Histology and histomorphometry of the fusion mass showed the formation of bone tissue in the fusion mass in both the BCP-EP/ABG and ABG groups, with the presence of typical bone morphology and bone-related cell types. Multinucleated cells were observed resorbing the BCP granules, which was responsible for the mode of action of the bone graft. The comparable fusion rate and bone formation results obtained in this study for BCP-EP/ABG and ABG and published for BCP granules in van Dijk et al. were expected since the same quantity of BCP granules was implanted [6]. The LEOL 400 polymer occupies intragranular space and clears rapidly to expose the needle-shaped surface topography typical of BCP granules that is at the origin of the mode of action of bone formation of the bone graft. The pro-healing capacity related to the needle-shaped surface topography was therefore preserved for BCP-EP.

## 5. Conclusions

The in vitro investigation of the degradation mechanism of BCP-EP and its bioactive properties showed that LEOL 400 hydrolyses in physiological conditions, resulting in unaltered PEG molecules, lactide species, and lactic acid, do not affect the chemical or physical composition of BCP granules. Moreover, 10 days of immersion in SBF highlighted that BCP-EP induces bone-like mineralization; thus, LEOL 400 does not affect the bioactivity of BCP granules in vitro. Finally, in vivo results from a rabbit PLF model demonstrated that rapid LEOL 400 resorption was evident as the BCP granules were exposed to cells and tissues after 3 days of implantation. Similar fusion rates were observed using different evaluation methods between ABG and BCP-EP/ABG after 12 weeks of implantation, demonstrating that the employment of BCP-EP/ABG results in solid fusion rates between the transverse processes. Furthermore, no migration of the granules or adverse reactions were noted, neither in the local area nor in distant organs. Hence, incorporating BCP granules in a novel and fast-resorbing LEOL 400 carrier does not affect the function of BCP granules both in vitro and in vivo, and the granules retain their bone-forming property. Driven by this evidence, we conclude that BCP-EP is a promising autograft extender for spinal fusion surgery.

## Figures and Tables

**Table 2:** Summary of fusion rates in rabbit PLF at 12 weeks post operation for BCP-EP/ABG and ABG.

Groups	Bilateral Fusion Manual Palpation	Fusion X-rays	Micro-CT Fusion	Histological Fusion
ABG	6 of 8	5 of 8 (Lenke A) 3 of 8 (Lenke B)	5 of 8 (Lenke A) 3 of 8 (Lenke B)	5 of 8
BCP-EP/ABG	6 of 8	6 of 8 (Lenke A) 2 of 8 (Lenke B)	6 of 8 (Lenke A) 2 of 8 (Lenke B)	6 of 8

## Data Availability

Data are contained within the article.
